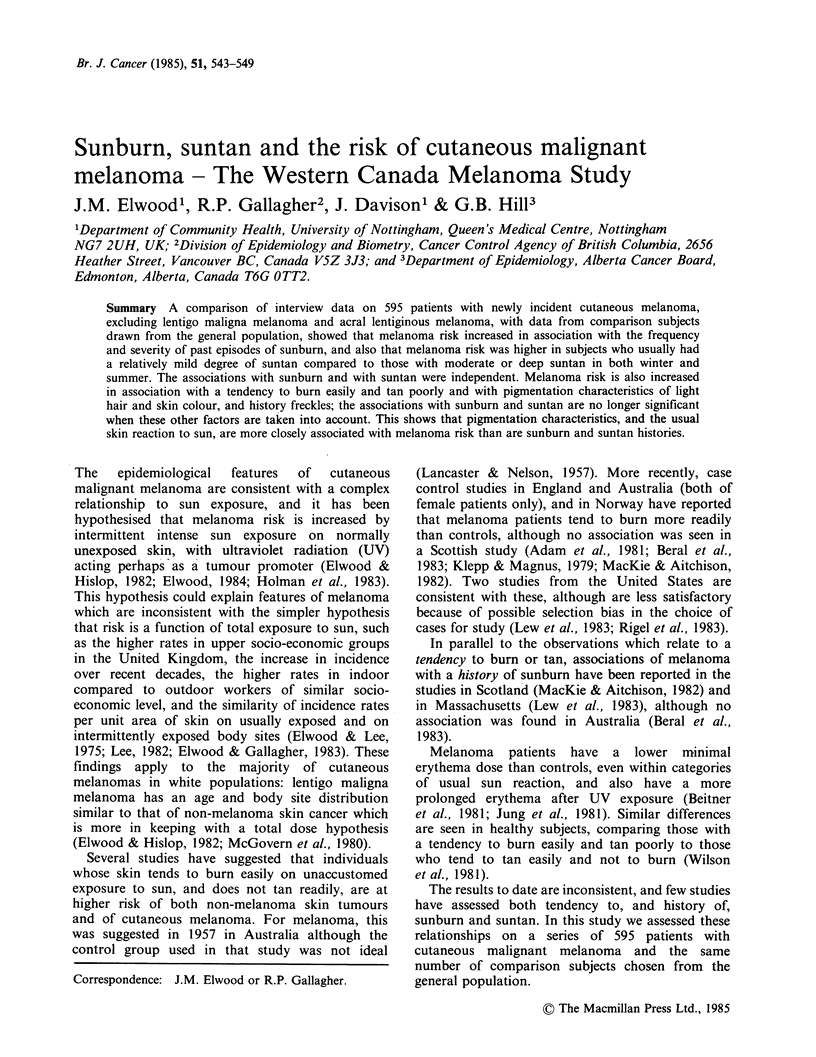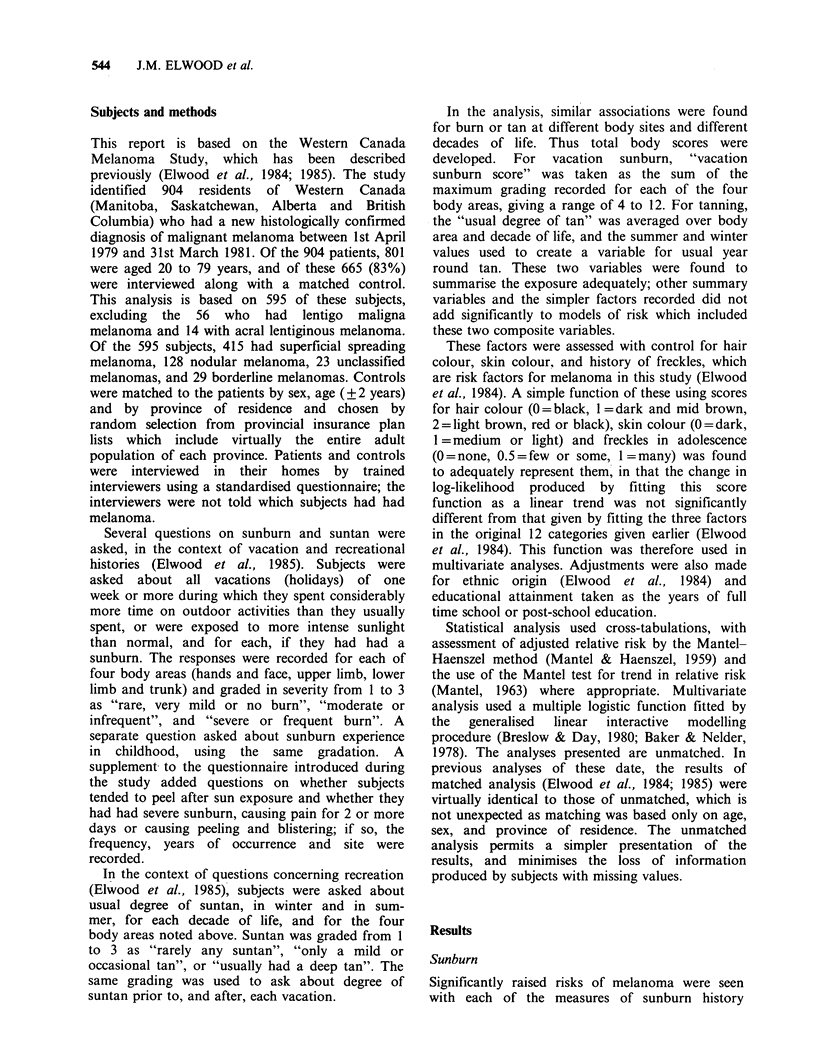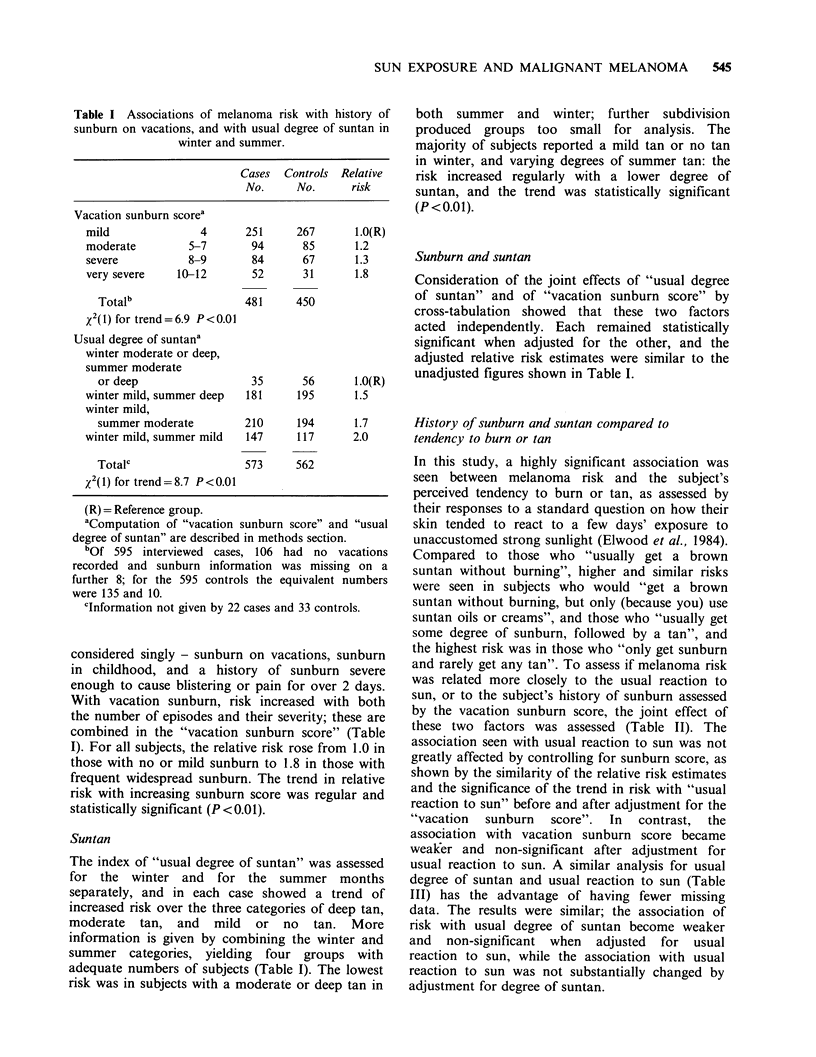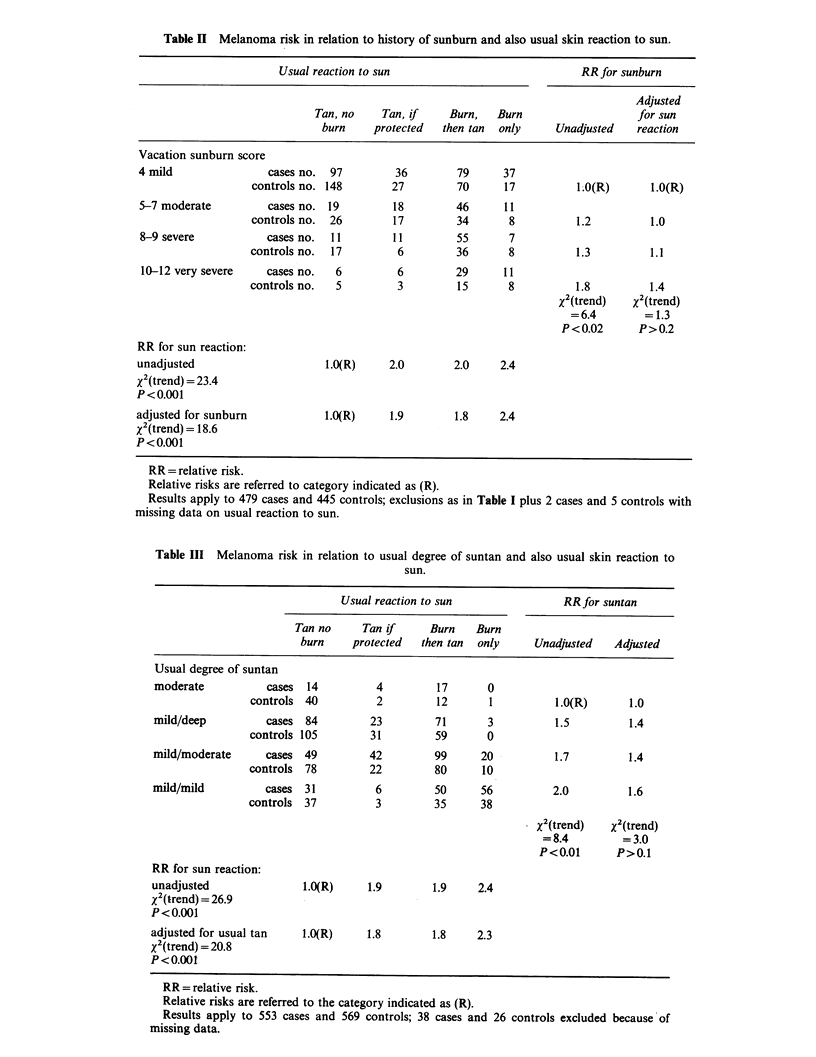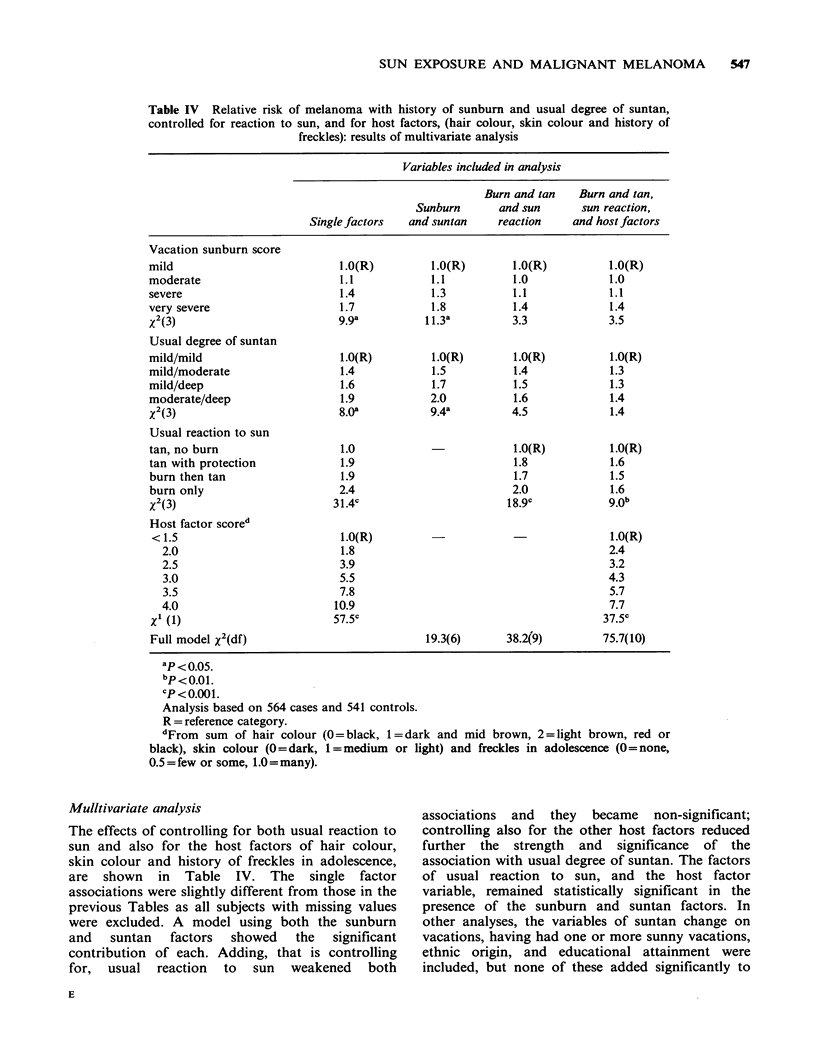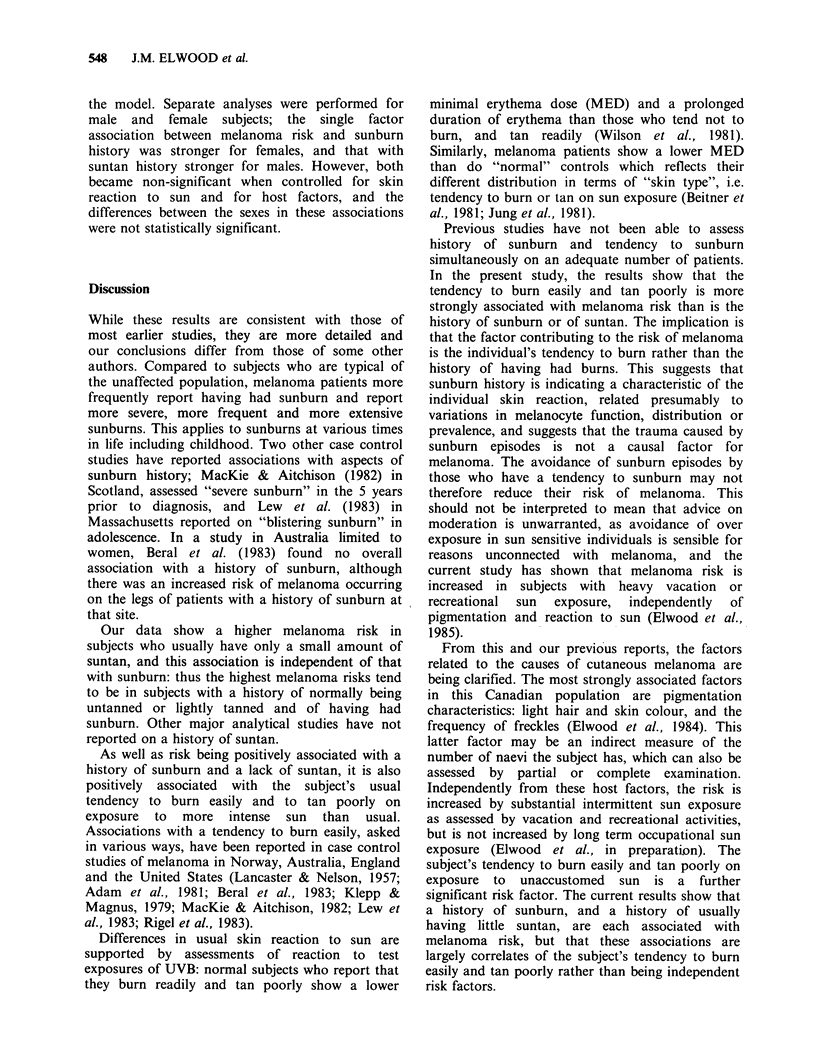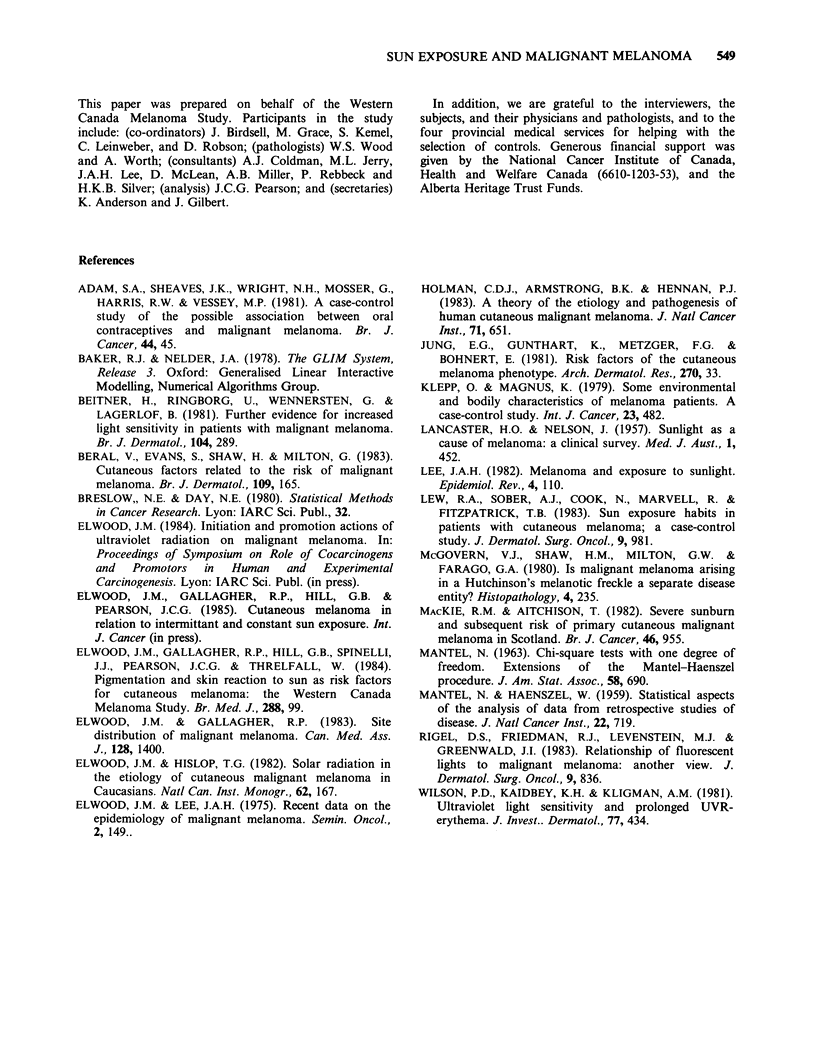# Sunburn, suntan and the risk of cutaneous malignant melanoma--The Western Canada Melanoma Study.

**DOI:** 10.1038/bjc.1985.77

**Published:** 1985-04

**Authors:** J. M. Elwood, R. P. Gallagher, J. Davison, G. B. Hill

## Abstract

A comparison of interview data on 595 patients with newly incident cutaneous melanoma, excluding lentigo maligna melanoma and acral lentiginous melanoma, with data from comparison subjects drawn from the general population, showed that melanoma risk increased in association with the frequency and severity of past episodes of sunburn, and also that melanoma risk was higher in subjects who usually had a relatively mild degree of suntan compared to those with moderate or deep suntan in both winter and summer. The associations with sunburn and with suntan were independent. Melanoma risk is also increased in association with a tendency to burn easily and tan poorly and with pigmentation characteristics of light hair and skin colour, and history freckles; the associations with sunburn and suntan are no longer significant when these other factors are taken into account. This shows that pigmentation characteristics, and the usual skin reaction to sun, are more closely associated with melanoma risk than are sunburn and suntan histories.


*Br. J. Cancer* (1985), **51**, 543–549

### Sunburn, suntan and the risk of cutaneous malignant melanoma – The Western Canada Melanoma Study

J.M. Elwood[1], R.P. Gallagher[2], J. Davison[1] & G.B. Hill[3]

[1]*Department of Community Health, University of Nottingham, Queen's Medical Centre, Nottingham NG7 2UH, UK;* [2]*Division of Epidemiology and Biometry, Cancer Control Agency of British Columbia, 2656 Heather Street, Vancouver BC, Canada V5Z 3J3; and* [3]*Department of Epidemiology, Alberta Cancer Board, Edmonton, Alberta, Canada T6G 0TT2.*

**Summary** A comparison of interview data on 595 patients with newly incident cutaneous melanoma, excluding lentigo maligna melanoma and acral lentiginous melanoma, with data from comparison subjects drawn from the general population, showed that melanoma risk increased in association with the frequency and severity of past episodes of sunburn, and also that melanoma risk was higher in subjects who usually had a relatively mild degree of suntan compared to those with moderate or deep suntan in both winter and summer. The associations with sunburn and with suntan were independent. Melanoma risk is also increased in association with a tendency to burn easily and tan poorly and with pigmentation characteristics of light hair and skin colour, and history freckles; the associations with sunburn and suntan are no longer significant when these other factors are taken into account. This shows that pigmentation characteristics, and the usual skin reaction to sun, are more closely associated with melanoma risk than are sunburn and suntan histories.

The epidemiological features of cutaneous malignant melanoma are consistent with a complex relationship to sun exposure, and it has been hypothesised that melanoma risk is increased by intermittent intense sun exposure on normally unexposed skin, with ultraviolet radiation (UV) acting perhaps as a tumour promoter (Elwood & Hislop, 1982; Elwood, 1984; Holman *et al.*, 1983). This hypothesis could explain features of melanoma which are inconsistent with the simpler hypothesis that risk is a function of total exposure to sun, such as the higher rates in upper socio-economic groups in the United Kingdom, the increase in incidence over recent decades, the higher rates in indoor compared to outdoor workers of similar socio-economic level, and the similarity of incidence rates per unit area of skin on usually exposed and on intermittently exposed body sites (Elwood & Lee, 1975; Lee, 1982; Elwood & Gallagher, 1983). These findings apply to the majority of cutaneous melanomas in white populations: lentigo maligna melanoma has an age and body site distribution similar to that of non-melanoma skin cancer which is more in keeping with a total dose hypothesis (Elwood & Hislop, 1982; McGovern *et al.*, 1980).

Several studies have suggested that individuals whose skin tends to burn easily on unaccustomed exposure to sun, and does not tan readily, are at higher risk of both non-melanoma skin tumours and of cutaneous melanoma. For melanoma, this was suggested in 1957 in Australia although the control group used in that study was not ideal (Lancaster & Nelson, 1957). More recently, case control studies in England and Australia (both of female patients only), and in Norway have reported that melanoma patients tend to burn more readily than controls, although no association was seen in a Scottish study (Adam *et al.*, 1981; Beral *et al.*, 1983; Klepp & Magnus, 1979; MacKie & Aitchison, 1982). Two studies from the United States are consistent with these, although are less satisfactory because of possible selection bias in the choice of cases for study (Lew *et al.*, 1983; Rigel *et al.*, 1983).

In parallel to the observations which relate to a *tendency* to burn or tan, associations of melanoma with a *history* of sunburn have been reported in the studies in Scotland (MacKie & Aitchison, 1982) and in Massachusetts (Lew *et al.*, 1983), although no association was found in Australia (Beral *et al.*, 1983).

Melanoma patients have a lower minimal erythema dose than controls, even within categories of usual sun reaction, and also have a more prolonged erythema after UV exposure (Beitner *et al.*, 1981; Jung *et al.*, 1981). Similar differences are seen in healthy subjects, comparing those with a tendency to burn easily and tan poorly to those who tend to tan easily and not to burn (Wilson *et al.*, 1981).

The results to date are inconsistent, and few studies have assessed both tendency to, and history of, sunburn and suntan. In this study we assessed these relationships on a series of 595 patients with cutaneous malignant melanoma and the same number of comparison subjects chosen from the general population.

Correspondence: J.M. Elwood or R.P. Gallagher.

© The Macmillan Press Ltd., 1985

### Subjects and methods

This report is based on the Western Canada Melanoma Study, which has been described previously (Elwood *et al.*, 1984; 1985). The study identified 904 residents of Western Canada (Manitoba, Saskatchewan, Alberta and British Columbia) who had a new histologically confirmed diagnosis of malignant melanoma between 1st April 1979 and 31st March 1981. Of the 904 patients, 801 were aged 20 to 79 years, and of these 665 (83%) were interviewed along with a matched control. This analysis is based on 595 of these subjects, excluding the 56 who had lentigo maligna melanoma and 14 with acral lentiginous melanoma. Of the 595 subjects, 415 had superficial spreading melanoma, 128 nodular melanoma, 23 unclassified melanomas, and 29 borderline melanomas. Controls were matched to the patients by sex, age ($\pm 2$ years) and by province of residence and chosen by random selection from provincial insurance plan lists which include virtually the entire adult population of each province. Patients and controls were interviewed in their homes by trained interviewers using a standardised questionnaire; the interviewers were not told which subjects had had melanoma.

Several questions on sunburn and suntan were asked, in the context of vacation and recreational histories (Elwood *et al.*, 1985). Subjects were asked about all vacations (holidays) of one week or more during which they spent considerably more time on outdoor activities than they usually spent, or were exposed to more intense sunlight than normal, and for each, if they had had a sunburn. The responses were recorded for each of four body areas (hands and face, upper limb, lower limb and trunk) and graded in severity from 1 to 3 as "rare, very mild or no burn", "moderate or infrequent", and "severe or frequent burn". A separate question asked about sunburn experience in childhood, using the same gradation. A supplement to the questionnaire introduced during the study added questions on whether subjects tended to peel after sun exposure and whether they had had severe sunburn, causing pain for 2 or more days or causing peeling and blistering; if so, the frequency, years of occurrence and site were recorded.

In the context of questions concerning recreation (Elwood *et al.*, 1985), subjects were asked about usual degree of suntan, in winter and in summer, for each decade of life, and for the four body areas noted above. Suntan was graded from 1 to 3 as "rarely any suntan", "only a mild or occasional tan", or "usually had a deep tan". The same grading was used to ask about degree of suntan prior to, and after, each vacation.

In the analysis, similar associations were found for burn or tan at different body sites and different decades of life. Thus total body scores were developed. For vacation sunburn, "vacation sunburn score" was taken as the sum of the maximum grading recorded for each of the four body areas, giving a range of 4 to 12. For tanning, the "usual degree of tan" was averaged over body area and decade of life, and the summer and winter values used to create a variable for usual year round tan. These two variables were found to summarise the exposure adequately; other summary variables and the simpler factors recorded did not add significantly to models of risk which included these two composite variables.

These factors were assessed with control for hair colour, skin colour, and history of freckles, which are risk factors for melanoma in this study (Elwood *et al.*, 1984). A simple function of these using scores for hair colour (0 = black, 1 = dark and mid brown, 2 = light brown, red or black), skin colour (0 = dark, 1 = medium or light) and freckles in adolescence (0 = none, 0.5 = few or some, 1 = many) was found to adequately represent them, in that the change in log-likelihood produced by fitting this score function as a linear trend was not significantly different from that given by fitting the three factors in the original 12 categories given earlier (Elwood *et al.*, 1984). This function was therefore used in multivariate analyses. Adjustments were also made for ethnic origin (Elwood *et al.*, 1984) and educational attainment taken as the years of full time school or post-school education.

Statistical analysis used cross-tabulations, with assessment of adjusted relative risk by the Mantel–Haenszel method (Mantel & Haenszel, 1959) and the use of the Mantel test for trend in relative risk (Mantel, 1963) where appropriate. Multivariate analysis used a multiple logistic function fitted by the generalised linear interactive modelling procedure (Breslow & Day, 1980; Baker & Nelder, 1978). The analyses presented are unmatched. In previous analyses of these date, the results of matched analysis (Elwood *et al.*, 1984; 1985) were virtually identical to those of unmatched, which is not unexpected as matching was based only on age, sex, and province of residence. The unmatched analysis permits a simpler presentation of the results, and minimises the loss of information produced by subjects with missing values.

### Results

### *Sunburn*

Significantly raised risks of melanoma were seen with each of the measures of sunburn history

**Table I** Associations of melanoma risk with history of sunburn on vacations, and with usual degree of suntan in winter and summer.

| | | Cases No. | Controls No. | Relative risk |
|---|---|---|---|---|
| Vacation sunburn score[a] | | | | |
| mild | 4 | 251 | 267 | 1.0(R) |
| moderate | 5–7 | 94 | 85 | 1.2 |
| severe | 8–9 | 84 | 67 | 1.3 |
| very severe | 10–12 | 52 | 31 | 1.8 |
| Total[b] | | 481 | 450 | |
| $\chi^2(1)$ for trend = 6.9 $P<0.01$ | | | | |
| Usual degree of suntan[a] | | | | |
| winter moderate or deep, summer moderate or deep | | 35 | 56 | 1.0(R) |
| winter mild, summer deep | | 181 | 195 | 1.5 |
| winter mild, summer moderate | | 210 | 194 | 1.7 |
| winter mild, summer mild | | 147 | 117 | 2.0 |
| Total[c] | | 573 | 562 | |
| $\chi^2(1)$ for trend = 8.7 $P<0.01$ | | | | |

(R) = Reference group.
[a]Computation of "vacation sunburn score" and "usual degree of suntan" are described in methods section.
[b]Of 595 interviewed cases, 106 had no vacations recorded and sunburn information was missing on a further 8; for the 595 controls the equivalent numbers were 135 and 10.
[c]Information not given by 22 cases and 33 controls.

considered singly – sunburn on vacations, sunburn in childhood, and a history of sunburn severe enough to cause blistering or pain for over 2 days. With vacation sunburn, risk increased with both the number of episodes and their severity; these are combined in the "vacation sunburn score" (Table I). For all subjects, the relative risk rose from 1.0 in those with no or mild sunburn to 1.8 in those with frequent widespread sunburn. The trend in relative risk with increasing sunburn score was regular and statistically significant ($P<0.01$).

*Suntan*

The index of "usual degree of suntan" was assessed for the winter and for the summer months separately, and in each case showed a trend of increased risk over the three categories of deep tan, moderate tan, and mild or no tan. More information is given by combining the winter and summer categories, yielding four groups with adequate numbers of subjects (Table I). The lowest risk was in subjects with a moderate or deep tan in both summer and winter; further subdivision produced groups too small for analysis. The majority of subjects reported a mild tan or no tan in winter, and varying degrees of summer tan: the risk increased regularly with a lower degree of suntan, and the trend was statistically significant ($P<0.01$).

*Sunburn and suntan*

Consideration of the joint effects of "usual degree of suntan" and of "vacation sunburn score" by cross-tabulation showed that these two factors acted independently. Each remained statistically significant when adjusted for the other, and the adjusted relative risk estimates were similar to the unadjusted figures shown in Table I.

*History of sunburn and suntan compared to tendency to burn or tan*

In this study, a highly significant association was seen between melanoma risk and the subject's perceived tendency to burn or tan, as assessed by their responses to a standard question on how their skin tended to react to a few days' exposure to unaccustomed strong sunlight (Elwood *et al.*, 1984). Compared to those who "usually get a brown suntan without burning", higher and similar risks were seen in subjects who would "get a brown suntan without burning, but only (because you) use suntan oils or creams", and those who "usually get some degree of sunburn, followed by a tan", and the highest risk was in those who "only get sunburn and rarely get any tan". To assess if melanoma risk was related more closely to the usual reaction to sun, or to the subject's history of sunburn assessed by the vacation sunburn score, the joint effect of these two factors was assessed (Table II). The association seen with usual reaction to sun was not greatly affected by controlling for sunburn score, as shown by the similarity of the relative risk estimates and the significance of the trend in risk with "usual reaction to sun" before and after adjustment for the "vacation sunburn score". In contrast, the association with vacation sunburn score became weaker and non-significant after adjustment for usual reaction to sun. A similar analysis for usual degree of suntan and usual reaction to sun (Table III) has the advantage of having fewer missing data. The results were similar; the association of risk with usual degree of suntan become weaker and non-significant when adjusted for usual reaction to sun, while the association with usual reaction to sun was not substantially changed by adjustment for degree of suntan.

**Table II** Melanoma risk in relation to history of sunburn and also usual skin reaction to sun.

| | | *Usual reaction to sun* | | | | *RR for sunburn* | |
|---|---|---|---|---|---|---|---|
| | | *Tan, no burn* | *Tan, if protected* | *Burn, then tan* | *Burn only* | *Unadjusted* | *Adjusted for sun reaction* |
| Vacation sunburn score | | | | | | | |
| 4 mild | cases no. | 97 | 36 | 79 | 37 | | |
| | controls no. | 148 | 27 | 70 | 17 | 1.0(R) | 1.0(R) |
| 5–7 moderate | cases no. | 19 | 18 | 46 | 11 | | |
| | controls no. | 26 | 17 | 34 | 8 | 1.2 | 1.0 |
| 8–9 severe | cases no. | 11 | 11 | 55 | 7 | | |
| | controls no. | 17 | 6 | 36 | 8 | 1.3 | 1.1 |
| 10–12 very severe | cases no. | 6 | 6 | 29 | 11 | | |
| | controls no. | 5 | 3 | 15 | 8 | 1.8 | 1.4 |
| | | | | | | $\chi^2$(trend) $=6.4$ $P<0.02$ | $\chi^2$(trend) $=1.3$ $P>0.2$ |
| RR for sun reaction: | | | | | | | |
| unadjusted $\chi^2$(trend)$=23.4$ $P<0.001$ | | 1.0(R) | 2.0 | 2.0 | 2.4 | | |
| adjusted for sunburn $\chi^2$(trend)$=18.6$ $P<0.001$ | | 1.0(R) | 1.9 | 1.8 | 2.4 | | |

RR = relative risk.
Relative risks are referred to category indicated as (R).
Results apply to 479 cases and 445 controls; exclusions as in **Table I** plus 2 cases and 5 controls with missing data on usual reaction to sun.

**Table III** Melanoma risk in relation to usual degree of suntan and also usual skin reaction to sun.

| | | *Usual reaction to sun* | | | | *RR for suntan* | |
|---|---|---|---|---|---|---|---|
| | | *Tan no burn* | *Tan if protected* | *Burn then tan* | *Burn only* | *Unadjusted* | *Adjusted* |
| Usual degree of suntan | | | | | | | |
| moderate | cases | 14 | 4 | 17 | 0 | | |
| | controls | 40 | 2 | 12 | 1 | 1.0(R) | 1.0 |
| mild/deep | cases | 84 | 23 | 71 | 3 | 1.5 | 1.4 |
| | controls | 105 | 31 | 59 | 0 | | |
| mild/moderate | cases | 49 | 42 | 99 | 20 | 1.7 | 1.4 |
| | controls | 78 | 22 | 80 | 10 | | |
| mild/mild | cases | 31 | 6 | 50 | 56 | 2.0 | 1.6 |
| | controls | 37 | 3 | 35 | 38 | | |
| | | | | | | $\chi^2$(trend) $=8.4$ $P<0.01$ | $\chi^2$(trend) $=3.0$ $P>0.1$ |
| RR for sun reaction: | | | | | | | |
| unadjusted $\chi^2$(trend)$=26.9$ $P<0.001$ | | 1.0(R) | 1.9 | 1.9 | 2.4 | | |
| adjusted for usual tan $\chi^2$(trend)$=20.8$ $P<0.001$ | | 1.0(R) | 1.8 | 1.8 | 2.3 | | |

RR = relative risk.
Relative risks are referred to the category indicated as (R).
Results apply to 553 cases and 569 controls; 38 cases and 26 controls excluded because of missing data.

**Table IV** Relative risk of melanoma with history of sunburn and usual degree of suntan, controlled for reaction to sun, and for host factors, (hair colour, skin colour and history of freckles): results of multivariate analysis

| | *Variables included in analysis* | | | |
|---|---|---|---|---|
| | *Single factors* | *Sunburn and suntan* | *Burn and tan and sun reaction* | *Burn and tan, sun reaction, and host factors* |
| Vacation sunburn score | | | | |
| mild | 1.0(R) | 1.0(R) | 1.0(R) | 1.0(R) |
| moderate | 1.1 | 1.1 | 1.0 | 1.0 |
| severe | 1.4 | 1.3 | 1.1 | 1.1 |
| very severe | 1.7 | 1.8 | 1.4 | 1.4 |
| $\chi^2(3)$ | 9.9[a] | 11.3[a] | 3.3 | 3.5 |
| Usual degree of suntan | | | | |
| mild/mild | 1.0(R) | 1.0(R) | 1.0(R) | 1.0(R) |
| mild/moderate | 1.4 | 1.5 | 1.4 | 1.3 |
| mild/deep | 1.6 | 1.7 | 1.5 | 1.3 |
| moderate/deep | 1.9 | 2.0 | 1.6 | 1.4 |
| $\chi^2(3)$ | 8.0[a] | 9.4[a] | 4.5 | 1.4 |
| Usual reaction to sun | | | | |
| tan, no burn | 1.0 | — | 1.0(R) | 1.0(R) |
| tan with protection | 1.9 | | 1.8 | 1.6 |
| burn then tan | 1.9 | | 1.7 | 1.5 |
| burn only | 2.4 | | 2.0 | 1.6 |
| $\chi^2(3)$ | 31.4[c] | | 18.9[c] | 9.0[b] |
| Host factor score[d] | | | | |
| <1.5 | 1.0(R) | — | — | 1.0(R) |
| 2.0 | 1.8 | | | 2.4 |
| 2.5 | 3.9 | | | 3.2 |
| 3.0 | 5.5 | | | 4.3 |
| 3.5 | 7.8 | | | 5.7 |
| 4.0 | 10.9 | | | 7.7 |
| $\chi^1$ (1) | 57.5[c] | | | 37.5[c] |
| Full model $\chi^2$(df) | | 19.3(6) | 38.2(9) | 75.7(10) |

[a]$P<0.05$.
[b]$P<0.01$.
[c]$P<0.001$.
Analysis based on 564 cases and 541 controls.
R = reference category.
[d]From sum of hair colour (0 = black, 1 = dark and mid brown, 2 = light brown, red or black), skin colour (0 = dark, 1 = medium or light) and freckles in adolescence (0 = none, 0.5 = few or some, 1.0 = many).

*Mulltivariate analysis*

The effects of controlling for both usual reaction to sun and also for the host factors of hair colour, skin colour and history of freckles in adolescence, are shown in Table IV. The single factor associations were slightly different from those in the previous Tables as all subjects with missing values were excluded. A model using both the sunburn and suntan factors showed the significant contribution of each. Adding, that is controlling for, usual reaction to sun weakened both associations and they became non-significant; controlling also for the other host factors reduced further the strength and significance of the association with usual degree of suntan. The factors of usual reaction to sun, and the host factor variable, remained statistically significant in the presence of the sunburn and suntan factors. In other analyses, the variables of suntan change on vacations, having had one or more sunny vacations, ethnic origin, and educational attainment were included, but none of these added significantly to the model. Separate analyses were performed for male and female subjects; the single factor association between melanoma risk and sunburn history was stronger for females, and that with suntan history stronger for males. However, both became non-significant when controlled for skin reaction to sun and for host factors, and the differences between the sexes in these associations were not statistically significant.

### Discussion

While these results are consistent with those of most earlier studies, they are more detailed and our conclusions differ from those of some other authors. Compared to subjects who are typical of the unaffected population, melanoma patients more frequently report having had sunburn and report more severe, more frequent and more extensive sunburns. This applies to sunburns at various times in life including childhood. Two other case control studies have reported associations with aspects of sunburn history; MacKie & Aitchison (1982) in Scotland, assessed "severe sunburn" in the 5 years prior to diagnosis, and Lew *et al.* (1983) in Massachusetts reported on "blistering sunburn" in adolescence. In a study in Australia limited to women, Beral *et al.* (1983) found no overall association with a history of sunburn, although there was an increased risk of melanoma occurring on the legs of patients with a history of sunburn at that site.

Our data show a higher melanoma risk in subjects who usually have only a small amount of suntan, and this association is independent of that with sunburn: thus the highest melanoma risks tend to be in subjects with a history of normally being untanned or lightly tanned and of having had sunburn. Other major analytical studies have not reported on a history of suntan.

As well as risk being positively associated with a history of sunburn and a lack of suntan, it is also positively associated with the subject's usual tendency to burn easily and to tan poorly on exposure to more intense sun than usual. Associations with a tendency to burn easily, asked in various ways, have been reported in case control studies of melanoma in Norway, Australia, England and the United States (Lancaster & Nelson, 1957; Adam *et al.*, 1981; Beral *et al.*, 1983; Klepp & Magnus, 1979; MacKie & Aitchison, 1982; Lew *et al.*, 1983; Rigel *et al.*, 1983).

Differences in usual skin reaction to sun are supported by assessments of reaction to test exposures of UVB: normal subjects who report that they burn readily and tan poorly show a lower minimal erythema dose (MED) and a prolonged duration of erythema than those who tend not to burn, and tan readily (Wilson *et al.*, 1981). Similarly, melanoma patients show a lower MED than do "normal" controls which reflects their different distribution in terms of "skin type", i.e. tendency to burn or tan on sun exposure (Beitner *et al.*, 1981; Jung *et al.*, 1981).

Previous studies have not been able to assess history of sunburn and tendency to sunburn simultaneously on an adequate number of patients. In the present study, the results show that the tendency to burn easily and tan poorly is more strongly associated with melanoma risk than is the history of sunburn or of suntan. The implication is that the factor contributing to the risk of melanoma is the individual's tendency to burn rather than the history of having had burns. This suggests that sunburn history is indicating a characteristic of the individual skin reaction, related presumably to variations in melanocyte function, distribution or prevalence, and suggests that the trauma caused by sunburn episodes is not a causal factor for melanoma. The avoidance of sunburn episodes by those who have a tendency to sunburn may not therefore reduce their risk of melanoma. This should not be interpreted to mean that advice on moderation is unwarranted, as avoidance of over exposure in sun sensitive individuals is sensible for reasons unconnected with melanoma, and the current study has shown that melanoma risk is increased in subjects with heavy vacation or recreational sun exposure, independently of pigmentation and reaction to sun (Elwood *et al.*, 1985).

From this and our previous reports, the factors related to the causes of cutaneous melanoma are being clarified. The most strongly associated factors in this Canadian population are pigmentation characteristics: light hair and skin colour, and the frequency of freckles (Elwood *et al.*, 1984). This latter factor may be an indirect measure of the number of naevi the subject has, which can also be assessed by partial or complete examination. Independently from these host factors, the risk is increased by substantial intermittent sun exposure as assessed by vacation and recreational activities, but is not increased by long term occupational sun exposure (Elwood *et al.*, in preparation). The subject's tendency to burn easily and tan poorly on exposure to unaccustomed sun is a further significant risk factor. The current results show that a history of sunburn, and a history of usually having little suntan, are each associated with melanoma risk, but that these associations are largely correlates of the subject's tendency to burn easily and tan poorly rather than being independent risk factors.

This paper was prepared on behalf of the Western Canada Melanoma Study. Participants in the study include: (co-ordinators) J. Birdsell, M. Grace, S. Kemel, C. Leinweber, and D. Robson; (pathologists) W.S. Wood and A. Worth; (consultants) A.J. Coldman, M.L. Jerry, J.A.H. Lee, D. McLean, A.B. Miller, P. Rebbeck and H.K.B. Silver; (analysis) J.C.G. Pearson; and (secretaries) K. Anderson and J. Gilbert.

In addition, we are grateful to the interviewers, the subjects, and their physicians and pathologists, and to the four provincial medical services for helping with the selection of controls. Generous financial support was given by the National Cancer Institute of Canada, Health and Welfare Canada (6610-1203-53), and the Alberta Heritage Trust Funds.

### References

ADAM, S.A., SHEAVES, J.K., WRIGHT, N.H., MOSSER, G., HARRIS, R.W. & VESSEY, M.P. (1981). A case-control study of the possible association between oral contraceptives and malignant melanoma. *Br. J. Cancer*, **44**, 45.

BAKER, R.J. & NELDER, J.A. (1978). *The GLIM System, Release 3*. Oxford: Generalised Linear Interactive Modelling, Numerical Algorithms Group.

BEITNER, H., RINGBORG, U., WENNERSTEN, G. & LAGERLOF, B. (1981). Further evidence for increased light sensitivity in patients with malignant melanoma. *Br. J. Dermatol.*, **104**, 289.

BERAL, V., EVANS, S., SHAW, H. & MILTON, G. (1983). Cutaneous factors related to the risk of malignant melanoma. *Br. J. Dermatol.*, **109**, 165.

BRESLOW,, N.E. & DAY, N.E. (1980). *Statistical Methods in Cancer Research*. Lyon: IARC Sci. Publ., **32**.

ELWOOD, J.M. (1984). Initiation and promotion actions of ultraviolet radiation on malignant melanoma. In: *Proceedings of Symposium on Role of Cocarcinogens and Promotors in Human and Experimental Carcinogenesis*. Lyon: IARC Sci. Publ. (in press).

ELWOOD, J.M., GALLAGHER, R.P., HILL, G.B. & PEARSON, J.C.G. (1985). Cutaneous melanoma in relation to intermittant and constant sun exposure. *Int. J. Cancer* (in press).

ELWOOD, J.M., GALLAGHER, R.P., HILL, G.B., SPINELLI, J.J., PEARSON, J.C.G. & THRELFALL, W. (1984). Pigmentation and skin reaction to sun as risk factors for cutaneous melanoma: the Western Canada Melanoma Study. *Br. Med. J.*, **288**, 99.

ELWOOD, J.M. & GALLAGHER, R.P. (1983). Site distribution of malignant melanoma. *Can. Med. Ass. J.*, **128**, 1400.

ELWOOD, J.M. & HISLOP, T.G. (1982). Solar radiation in the etiology of cutaneous malignant melanoma in Caucasians. *Natl Can. Inst. Monogr.*, **62**, 167.

ELWOOD, J.M. & LEE, J.A.H. (1975). Recent data on the epidemiology of malignant melanoma. *Semin. Oncol.*, **2**, 149..

HOLMAN, C.D.J., ARMSTRONG, B.K. & HENNAN, P.J. (1983). A theory of the etiology and pathogenesis of human cutaneous malignant melanoma. *J. Natl Cancer Inst.*, **71**, 651.

JUNG, E.G., GUNTHART, K., METZGER, F.G. & BOHNERT, E. (1981). Risk factors of the cutaneous melanoma phenotype. *Arch. Dermatol. Res.*, **270**, 33.

KLEPP, O. & MAGNUS, K. (1979). Some environmental and bodily characteristics of melanoma patients. A case-control study. *Int. J. Cancer*, **23**, 482.

LANCASTER, H.O. & NELSON, J. (1957). Sunlight as a cause of melanoma: a clinical survey. *Med. J. Aust.*, **1**, 452.

LEE, J.A.H. (1982). Melanoma and exposure to sunlight. *Epidemiol. Rev.*, **4**, 110.

LEW, R.A., SOBER, A.J., COOK, N., MARVELL, R. & FITZPATRICK, T.B. (1983). Sun exposure habits in patients with cutaneous melanoma; a case-control study. *J. Dermatol. Surg. Oncol.*, **9**, 981.

McGOVERN, V.J., SHAW, H.M., MILTON, G.W. & FARAGO, G.A. (1980). Is malignant melanoma arising in a Hutchinson's melanotic freckle a separate disease entity? *Histopathology*, **4**, 235.

MACKIE, R.M. & AITCHISON, T. (1982). Severe sunburn and subsequent risk of primary cutaneous malignant melanoma in Scotland. *Br. J. Cancer*, **46**, 955.

MANTEL, N. (1963). Chi-square tests with one degree of freedom. Extensions of the Mantel–Haenszel procedure. *J. Am. Stat. Assoc.*, **58**, 690.

MANTEL, N. & HAENSZEL, W. (1959). Statistical aspects of the analysis of data from retrospective studies of disease. *J. Natl Cancer Inst.*, **22**, 719.

RIGEL, D.S., FRIEDMAN, R.J., LEVENSTEIN, M.J. & GREENWALD, J.I. (1983). Relationship of fluorescent lights to malignant melanoma: another view. *J. Dermatol. Surg. Oncol.*, **9**, 836.

WILSON, P.D., KAIDBEY, K.H. & KLIGMAN, A.M. (1981). Ultraviolet light sensitivity and prolonged UVR-erythema. *J. Invest.. Dermatol.*, **77**, 434.